# Effectiveness of pharmaceutical strategies for pandemic H1N1 2009 influenza in TAIWAN

**DOI:** 10.1186/1753-6561-5-S6-P88

**Published:** 2011-06-29

**Authors:** U-I Wu, F-C Hu, S-C Chang, W-R Lin, M-C Lu, P-L Lu, Y-C Chen

**Affiliations:** 1 National Taiwan University Hospital, Taipei, China; 2 National Taiwan University College of Medicine, Taipei, China; 3 Kaohsiung Medical University Hospital, Kaoshiung, Taiwan, China; 4 Chung-Shan Medical University Hospital, Taichung, Taiwan, China

## Introduction / objectives

Mass vaccination campaign conducted according to the priority groups, and provision of free rapid influenza diagnostic tests (RIDTs) and antivirals to predefined population at flu clinics, were implemented as a national program in Taiwan to reduce the impacts of the pandemic H1N1 2009 (pH1N1) influenza. This multi-center study aimed to evaluate the effectiveness of these pharmaceutical interventions based on hospital-wide influenza surveillance data.

## Methods

From 15 Aug 2009 through 1 March 2010, all in- and outpatients who received RIDTs at 3 teaching hospitals located in the northern, middle, and southern Taiwan, respectively were analyzed. A time-series analysis was conducted to estimate the effects of various pharmaceutical strategies on the number of patients with positive RIDTs each day. The daily mean level of population immunity was estimated based on the nationwide vaccination coverage rate, seroconversion rate in different age groups and simulated time-lag for seroconversion.

## Results

A total of 7,206 out of 34,359 patients had positive RIDT results for influenza A. The greatest number of daily positive cases in each hospital ranged from 40 to 56, and there were slight regional differences regarding the profile of daily positive rates. Multivariate analysis of the data from the largest hospital showed that establishment of flu clinics averagely reduce 7 daily cases (*p* < 0.001), and an increment of 10% daily mean level of population immunity against pH1N1 through vaccination averagely reduce 5 daily cases (*p* < 0.001). Similar results were obtained in the other two hospitals.

## Conclusion

Pharmaceutical strategies implemented in Taiwan were effective in lowering the burden of pH1N1 influenza.

## Disclosure of interest

None declared.

